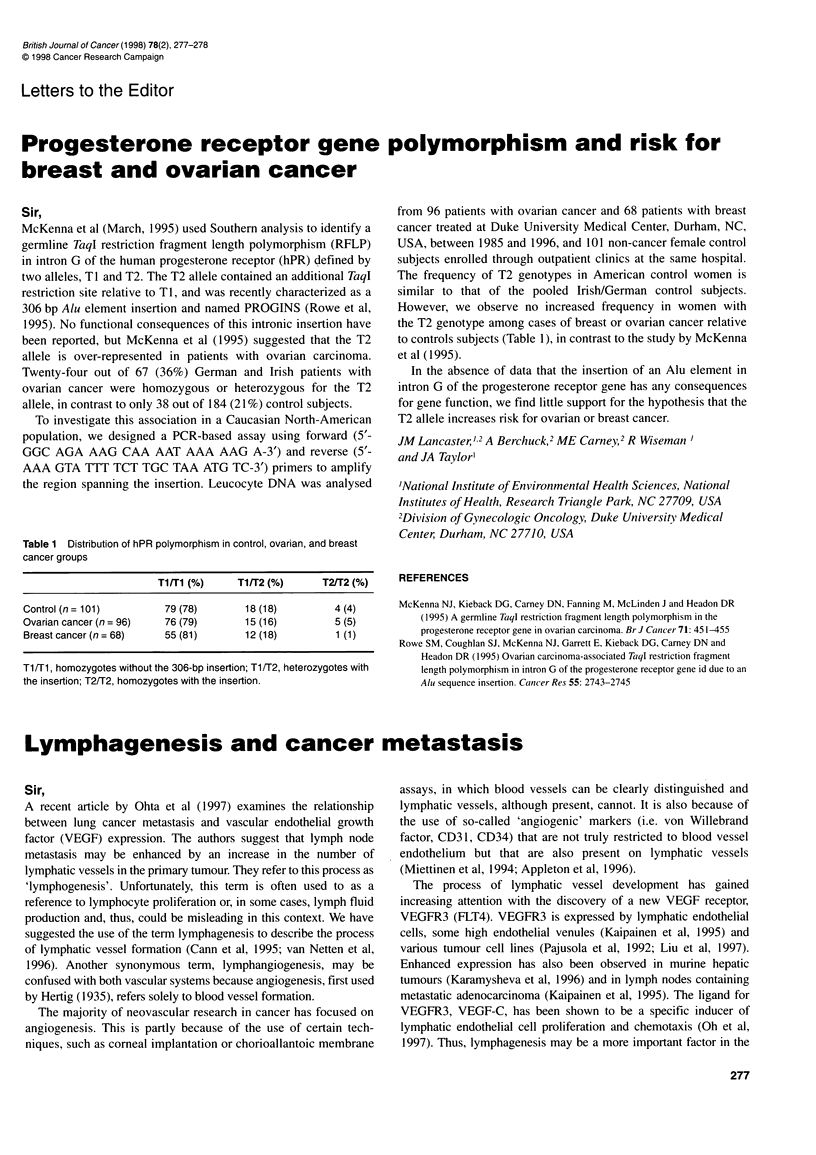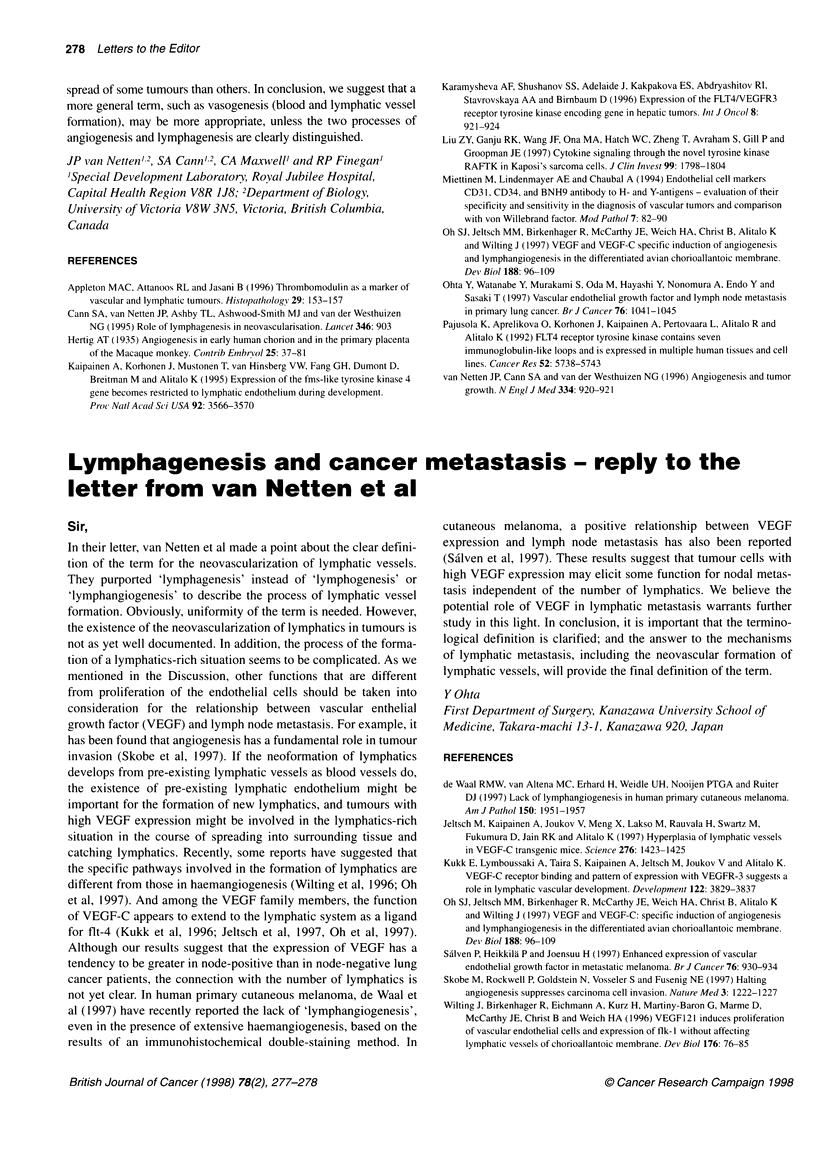# Lymphagenesis and cancer metastasis.

**DOI:** 10.1038/bjc.1998.481

**Published:** 1998-07

**Authors:** J. P. van Netten, S. A. Cann, C. A. Maxwell, R. P. Finegan


### Lymphagenesis and cancer metastasis

**Sir,**

A recent article by Ohta et al (1997) examines the relationship between lung cancer metastasis and vascular endothelial growth factor (VEGF) expression. The authors suggest that lymph node metastasis may be enhanced by an increase in the number of lymphatic vessels in the primary tumour. They refer to this process as ‘lymphogenesis’. Unfortunately, this term is often used to as a reference to lymphocyte proliferation or, in some cases, lymph fluid production and, thus, could be misleading in this context. We have suggested the use of the term lymphagenesis to describe the process of lymphatic vessel formation (Cann et al, 1995; van Netten et al, 1996). Another synonymous term, lymphangiogenesis, may be confused with both vascular systems because angiogenesis, first used by Hertig (1935), refers solely to blood vessel formation.

The majority of neovascular research in cancer has focused on angiogenesis. This is partly because of the use of certain techniques, such as corneal implantation or chorioallantoic membrane assays, in which blood vessels can be clearly distinguished and lymphatic vessels, although present, cannot. It is also because of the use of so-called ‘angiogenic’ markers (i.e. von Willebrand factor, CD31, CD34) that are not truly restricted to blood vessel endothelium but that are also present on lymphatic vessels (Miettinen et al, 1994; Appleton et al, 1996).

The process of lymphatic vessel development has gained increasing attention with the discovery of a new VEGF receptor, VEGFR3 (FLT4). VEGFR3 is expressed by lymphatic endothelial cells, some high endothelial venules (Kaipainen et al, 1995) and various tumour cell lines (Pajusola et al, 1992; Liu et al, 1997). Enhanced expression has also been observed in murine hepatic tumours (Karamysheva et al, 1996) and in lymph nodes containing metastatic adenocarcinoma (Kaipainen et al, 1995). The ligand for VEGFR3, VEGF-C, has been shown to be a specific inducer of lymphatic endothelial cell proliferation and chemotaxis (Oh et al, 1997). Thus, lymphagenesis may be a more important factor in the spread of some tumours than others. In conclusion, we suggest that a more general term, such as vasogenesis (blood and lymphatic vessel formation), may be more appropriate, unless the two processes of angiogenesis and lymphagenesis are clearly distinguished.

*JP van Netten[1,2], SA Cann[1,2], CA Maxwell[1] and RP Finegan[1]*

*[1]Special Development Laboratory, Royal Jubilee Hospital, Capital Health Region V8R 1J8; [2]Department of Biology, University of Victoria V8W 3N5, Victoria, British Columbia, Canada*

**REFERENCES**

Appleton MAC, Attanoos RL and Jasani B (1996) Thrombomodulin as a marker of vascular and lymphatic tumours. *Histopathology* **29**: 153–157

Cann SA, van Netten JP, Ashby TL, Ashwood-Smith MJ and van der Westhuizen NG (1995) Role of lymphagenesis in neovascularisation. *Lancet* **346**: 903

Hertig AT (1935) Angiogenesis in early human chorion and in the primary placenta of the Macaque monkey. *Contrib Embryol* **25**: 37–81

Kaipainen A, Korhonen J, Mustonen T, van Hinsberg VW, Fang GH, Dumont D, Breitman M and Alitalo K (1995) Expression of the fms-like tyrosine kinase 4 gene becomes restricted to lymphatic endothelium during development. *Proc Natl Acad Sci USA* **92**: 3566–3570

Karamysheva AF, Shushanov SS, Adelaide J, Kakpakova ES, Abdryashitov RI, Stavrovskaya AA and Birnbaum D (1996) Expression of the FLT4/VEGFR3 receptor tyrosine kinase encoding gene in hepatic tumors. *Int J Oncol* **8**: 921–924

Liu ZY, Ganju RK, Wang JF, Ona MA, Hatch WC, Zheng T, Avraham S, Gill P and Groopman JE (1997) Cytokine signaling through the novel tyrosine kinase RAFTK in Kaposi's sarcoma cells. *J Clin Invest* **99**: 1798–1804

Miettinen M, Lindenmayer AE and Chaubal A (1994) Endothelial cell markers CD31, CD34, and BNH9 antibody to H- and Y-antigens – evaluation of their specificity and sensitivity in the diagnosis of vascular tumors and comparison with von Willebrand factor. *Mod Pathol* **7**: 82–90

Oh SJ, Jeltsch MM, Birkenhager R, McCarthy JE, Weich HA, Christ B, Alitalo K and Wilting J (1997) VEGF and VEGF-C specific induction of angiogenesis and lymphangiogenesis in the differentiated avian chorioallantoic membrane. *Dev Biol* **188**: 96–109

Ohta Y, Watanabe Y, Murakami S, Oda M, Hayashi Y, Nonomura A, Endo Y and Sasaki T (1997) Vascular endothelial growth factor and lymph node metastasis in primary lung cancer. *Br J Cancer* **76**: 1041–1045

Pajusola K, Aprelikova O, Korhonen J, Kaipainen A, Pertovaara L, Alitalo R and Alitalo K (1992) FLT4 receptor tyrosine kinase contains seven immunoglobulin-like loops and is expressed in multiple human tissues and cell lines. *Cancer Res* **52**: 5738–5743

van Netten JP, Cann SA and van der Westhuizen NG (1996) Angiogenesis and tumor growth. *N Engl J Med* **334**: 920–921